# The Social Ecological Model: A Framework for Understanding COVID-19 Vaccine Uptake among Healthcare Workers—A Scoping Review

**DOI:** 10.3390/vaccines11091491

**Published:** 2023-09-15

**Authors:** Damian Naidoo, Anna Meyer-Weitz, Kaymarlin Govender

**Affiliations:** 1 Discipline of Psychology, School of Applied Human Sciences, Howard College, University of KwaZulu-Natal, Durban 4041, South Africa; 2 Health Promotion Unit, KwaZulu-Natal Department of Health, Pietermaritzburg, Private Bag X9051, Pietermaritzburg 3200, South Africa; 3 HEARD, College of Law and Management Studies, University of Kwazulu-Natal, Durban 4041, South Africa

**Keywords:** HCWs, COVID-19 vaccines, Africa, Social Ecological Model, barriers, facilitators, scoping review

## Abstract

Vaccination plays a crucial role in combating the global COVID-19 pandemic. Immunizing all healthcare workers (HCWs) is essential for increasing vaccine confidence and acceptance within the general population. Understanding the factors that hinder or facilitate vaccine uptake among HCWs is of utmost importance, considering they are among the first to be vaccinated. This review follows Arksey and O’Malley’s five-stage methodological framework. We searched PubMed, Web of Science, ProQuest, WorldCat Discovery, and Google Scholar for peer-reviewed articles published from 2020 to 2023. A descriptive analysis and narrative synthesis approach were employed to collect and synthesize data. Using the social-ecological model as a framework, the literature was categorized into themes at the intrapersonal, interpersonal, organizational, community, and policy levels. We reviewed a total of fifty-three published academic articles, with the majority of studies conducted in Ethiopia and Nigeria. The intention for vaccine uptake resulted in an unsatisfactory (52%) overall uptake rate among HCWs. Individual-level determinants associated with vaccine uptake included being male, middle-aged, being a physician, having a higher level of education, and having a chronic illness. This review identified significant barriers at each level, such as safety concerns, perceived scientific uncertainty, vaccine ineffectiveness, lack of trust in stakeholders, and religious beliefs. Additionally, we identified facilitators at each level, with the most common factors promoting intention to uptake being the desire to protect oneself and others and a high perceived susceptibility to contracting COVID-19. This review highlights the existence of significant barriers to vaccine uptake on the African continent. Given that HCWs play a crucial role in guiding the public’s vaccination decisions, it is imperative to prioritize education and training efforts about the safety and effectiveness of COVID-19 vaccines.

## 1. Introduction

The World Health Organization (WHO) approved several vaccines against COVID-19 for global distribution in various regions [1,2]. Vaccines manufactured by Pfizer, Oxford/AstraZeneca, Moderna, Janssen, Sputnik V, Sinovac, and Sinopharm, among others, were authorized and made available in Africa [2,3]. In the first quarter of 2021, mass vaccination programs commenced in several African countries [2,3,4]. These campaigns were planned in 31 African countries until 2022 [5]. Egypt was the first African country to begin vaccination on 24 January 2021, followed by South Africa on 17 February 2021, and Zimbabwe on 18 February 2021 [4]. During the distribution of the COVID-19 vaccination, there have been substantial problems with vaccine nationalism and access equity [6] Hence, Africa and other low-and middle-income countries (LMICs) have low COVID-19 vaccine coverage [7]. As a result, the COVAX global initiative was established to ensure equitable and timely access to vaccines worldwide [8]. The continent received more than 892 million vaccine doses, with the COVAX facility accounting for 64% of the total vaccinations received [9]. Much progress has been made in increasing vaccine shipments to countries [10,11]. Despite greater access to COVID-19 vaccinations, the COVID-19 pandemic has exposed numerous flaws in African healthcare systems, particularly in the aftermath of the Delta and Omicron variants [10,12]. As of 16 October 2022, only 24% of the African continent’s population had been vaccinated, compared to a global coverage of 64% [13]. According to the WHO, Africa is on track to reach the global vaccination coverage target of 70% by April 2025 [13]. As vaccine supply has increased worldwide, it has become clear that COVID-19 vaccine hesitancy (VH) challenges vaccine uptake [14,15] in Africa [8,16], particularly in Western and Central Africa [17]. The WHO ranked VH as one of the top ten threats to global health [14,16] and defines it as “a delay in acceptance or refusal of vaccines despite availability of vaccination services” [18] (p. 899). This broad definition highlights variability by stating that VH varies between vaccine types, contexts, geographical regions, and over time. This phenomenon has been exacerbated by the current COVID-19 pandemic [15,19].

Due to a global shortage of COVID-19 vaccines, governments have prioritized high-risk groups for vaccination [11,20,21]. Despite African countries prioritizing healthcare workers (HCWs), vaccine coverage remains low due to VH and a lack of vaccination services and fear of its side effects, especially in rural areas, leaving the vast majority of front-line workers unprotected [4,11,22]. Studies showed that not all HCWs are prepared to receive the COVID-19 vaccine when it becomes available in their country [8,22,23]. Concerns have been raised about VH among HCWs throughout Africa [11,22]. Vaccine acceptance (VA) and hesitancy have been a global problem, particularly in African settings [16,24,25]. Historical, structural, and other systemic dynamics contribute to VH in the African continent [7,8], and are a remaining threat to Africa’s vaccination programmes [17]. The increased polio outbreaks in Nigeria have been argued to stem from misinformation and public distrust in vaccination between 2002 and 2006 and subsequent polio outbreaks on three continents [8,26]. Furthermore, mass deworming programmes in Ghana were rejected due to community misconceptions [8]. Furthermore, trust in current vaccines has been eroded by a history of colonial medical and vaccine research abuse in Africa [7]. African populations were frequently subjected to unethical testing in the name of scientific advancement [7,27]. At the beginning of 2021, Tanzania’s health minister announced that the country would forgo COVID-19 vaccination due to concerns about vaccine safety and would instead depend on traditional and household herbs and medicines for prevention and cure [28,29].

There are numerous barriers and drivers that influence vaccination intention (VI) and uptake, ranging from individual psychological, socio-cultural, and environmental factors that influence HCW’s willingness to be vaccinated [30,31,32,33]. The Social Ecological Model (SEM) was initially developed by Urie Bronfenbrenner [34] and later adapted by McLeroy and colleagues [35]. This framework, widely used in public health and social sciences, aims to comprehend the various factors influencing human behaviour and health outcomes [34,35]. It acknowledges that individuals exist within different social systems and that multiple levels of influence interact to shape their behaviours [35]. These levels are as follows: Intrapersonal Level: this level focuses on the characteristics and attributes of individuals, including factors such as knowledge, attitudes, beliefs, skills, and biological factors. Interpersonal Level: The interpersonal level involves the impact of relationships and social networks on an individual. It includes family, friends, peers, co-workers, and other social connections. Organizational Level: The organizational level pertains to formal and informal rules, policies, and practices. It can encompass schools, workplaces, community organizations, and religious institutions. Organizational factors can affect access to resources, opportunities, and social norms. Community Level: The community level encompasses the physical and social environment in which individuals reside. It includes the characteristics of the community, such as its infrastructure, social capital, and cultural norms. Community factors can influence social norms, social networks, and the availability of resources and services. Policy Level: The policy level represents the broader social, economic, and political context in which individuals and communities are situated. It encompasses public policies, laws, social inequality, and cultural values.

In light of continuous COVID-19 infections and the likelihood of future pandemics, HCW’s hesitation in vaccination uptake remains an area of concern. Given that HCWs are among the first to be vaccinated, it is critical to understand factors that pose barriers or facilitate vaccine uptake. In light of this, we used the five-level SEM to segment the levels of influence (intrapersonal, interpersonal, organizational, community, and policy level) to provide a more comprehensive and nuanced understanding of how these factors shape vaccine-related behaviours. The identified factors were organized into barriers and facilitators to clarify their influence on VA and VH. While a review had been conducted on VA on the African continent among HCWs [36], this review focused on factors and barriers influencing COVID-19 vaccine acceptance, intention for uptake, and hesitancy among HCWs on the African continent in lieu of informing intervention approaches to address likely barriers in future immunization programmes.

## 2. Methods

This scoping review was conducted using Arksey and O’Malley’s methodological framework [37]. The following five-stage framework proposed was as follows: “(1) Identifying the research questions, (2) Searching for relevant studies, (3) Selecting studies, (4) Charting the data, and (5) Collating, summarising, and reporting the results” [37] (p. 22). This review includes the Preferred Reporting Items for Systematic Review and Meta-Analysis extension for Scoping Reviews (PRISMA-ScR) checklist (Supplementary Materials S1) [38]. A review protocol was submitted to the University of KwaZulu-Natal (UKZN) Humanities and Social Sciences Research Ethics Committee (HSSREC)—Application number: 00013262.

### 2.1. Identifying the Research Questions

1. What is the rate of uptake of COVID-19 vaccinations among HCWs?
2. What socio-demographic factors are associated with VA or VH among HCWs?
3. What factors act as barriers or facilitators for vaccine uptake among HCWs?

### 2.2. Searching for Relevant Studies

A comprehensive literature search was conducted in five databases: Web of Science, WorldCat Discovery, PubMed, Google Scholar, and ProQuest to retrieve studies related to the above research questions, and the search period for the review spanned from 2020 to 2023. The final search was completed in May 2023. The COVID-19 pandemic was the motivating factor behind this timeline. The following search terms were applied, using a variation of MEsH terms and keywords for each database: “COVID-19 vaccines”, “COVID-19”, “SARS-CoV-2 vaccines”, “associated factors”, “intention”, “barriers”, “drivers”, “acceptance”, “hesitancy”, “Africa”, “Healthcare workers”, “vaccine uptake”, “vaccine refusal”, “HCWs”, “COVID-19 vaccination uptake”, “COVID-19 vaccination intention”, “COVID-19 vaccine willingness”. The final search strategies for WorldCat Discovery and PubMed are in Appendix A, Table A1, Table A2 and Table A3.

### 2.3. Study Selection

After thoroughly screening the titles and abstracts, inclusion and exclusion criteria were established initially and studies were considered using the Population–Concept–Context (PCC) framework to determine their eligibility for this review. Full-text eligible studies met the following inclusion criteria: (1) literature type: academic/published journals (peer-reviewed journals); (2) language: studies that were published in the English language; (3) timeline: studies that were published between 2021 and 2023, (4) location: studies conducted in Africa; (5) vaccines: COVID-19 vaccines; (6) populations: HCWs—using the WHO definition of HCWs [39] (7) study designs: quantitative, qualitative, or mix-methods studies; (8) studies that specifically address the research questions. The following were excluded: grey literature (unpublished journals, reports and documents, conference papers, memoranda, theses, letters, and protocols) and reviews (scoping and systematic).

### 2.4. Charting Data

Data extraction from the included peer-reviewed studies was conducted using a standardized Microsoft Excel data collection sheet. A reviewer (D.N) extracted data from included reviews, which was then independently verified by a second reviewer (A.M-W). The following data fields were extracted from each study: author, year of publication, country, data collection period, methodology and study design, population characteristics, sample size, and measurement scales. The VI, VH, and VA levels among HCWs were analysed, summarised, and compared using simple descriptive statistics (percentages). A narrative synthesis approach [40] was utilized to acquire, synthesize, and map the literature utilizing the SEM to group facilitators and barriers to the uptake of the COVID-19 vaccine. All data were reported using thematic narratives [41].

### 2.5. Collating, Summarising, and Reporting the Results

The results have been compiled and summarized. Following a description of the study’s characteristics, the relevant influencing factors are presented using the SEM. Barriers and facilitators impacting the uptake of COVID-19 vaccines were categorized into various levels, including socio-demographic characteristics, individual factors, social factors, institutional factors, community factors, and policy factors.

## 3. Results

**Figure 1:**
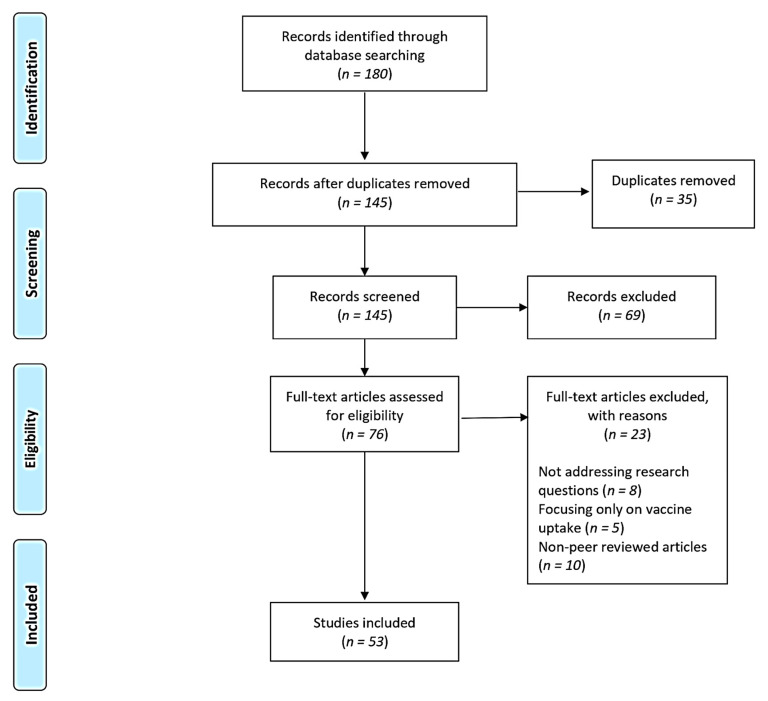
PRISMA flow diagram: selection of included studies. Adapted from [42].

A total of 180 records were identified from the five database searches: Web of Science (*n* = 20), WorldCat Discovery (*n* = 16), PubMed (*n* = 41), Google Scholar (*n* = 55) and ProQuest (*n* = 48). After removing duplicates using EndNote (V.X9), 145 records remained for a title and abstract screening. We excluded 69 articles that did not meet the selection criteria, leaving 76 for a review of the full-text articles. The full-text screening was conducted to assess eligibility before further data extraction. Following the inclusion and exclusion assessment criteria, studies were further excluded because they did not address research questions (*n* = 8), focused solely on vaccine uptake (*n* = 5), and were non-peer-reviewed (*n* = 10), resulting in 53 articles included in the final review. The PRISMA flow diagram below illustrates the selection process in Figure 1.

### 3.1. Descriptive Analysis of Articles

The majority of the articles included in this review were conducted in Ethiopia (23%), followed by Nigeria (17%), Egypt (13%), South Africa (8%), and Ghana (8%). The remaining articles were conducted in Cameroon, Uganda, Somalia, Tanzania, Namibia, Malawi, Zambia, The Democratic Republic of Congo (DRC), Guinea, Sudan, Sierra Leone, and Tunisia. Two articles focused on multiple African countries, including Nigeria, Cameroon, Sierra Leone, DRC, and Uganda. Please refer to Table 1 for the number of countries reviewed and Appendix B, Table A4 for the included study characteristics.

The majority of the studies used a quantitative cross-sectional design (88%), while six studies employed a mixed-method design (8%), and one used a qualitative design (4%). This review specifically focused on HCWs, with the exception of a study conducted by Toure and colleagues [43], which also surveyed the general adult population. Since this review had specific exclusion criteria, only the sampled population of HCWs was considered. The sample size of the included studies varied from 15 to 7763 participants. Among the sampled HCWs, the majority were physicians (83%), followed by nurses (73%), pharmacists (49%), medical laboratory technicians (47%), and midwives (42%).

### 3.2. Survey Instruments/Measurement Scales

There are various types of measurement scales or survey instruments used in research. The articles reviewed in this study employed two types of measurement scales, dichotomous scales and Likert scales, to assess VH or VA. A dichotomous question presents only two possible answer options [44]. This type of question is considered closed-ended because the options are predetermined by the investigator. Dichotomous questions are used when there are only two possible values for the subject being examined [44]. On the other hand, a Likert scale is a rating scale used to evaluate opinions, attitudes, or behaviours. It consists of a statement or question followed by a set of answer statements, typically five, seven, or nine in number [45].

In this review, 12 studies utilized Likert scales, while 36 studies utilized dichotomous scales to measure vaccine uptake. Upon screening the articles, variations in measurement approaches were identified. For example, authors assessed VH or VA using a Likert scale in the following ways. El-Sokkary and colleagues [46] measured vaccination intention by asking participants to indicate their intention to undergo COVID-19 vaccination on a three-point scale: “agree”, “neutral”, or “disagree”. Fares and colleagues [47] measured the decision to receive the COVID-19 vaccine with three options: “yes”, “no”, or “undecided”. In their study, the term “hesitant” was used for the undecided group. Wiysonge and colleagues [48] assessed vaccine acceptance by using the statement, “I will take the COVID-19 vaccine when one becomes available”. This statement had seven response options ranging from “strongly disagree” to “strongly agree”. The responses were later transformed into a binary variable, with responses 1 to 4 categorized as “vaccine hesitancy” and responses 5 to 7 categorized as “vaccine acceptance”.

In terms of dichotomous scales, VH and VA were assessed as follows, Adejumo and colleagues [49] evaluated participants’ willingness to receive the COVID-19 vaccine using single-item questions with “yes” or “no” responses. Yilma and colleagues [50] assessed vaccine acceptability by asking, “If a COVID-19 vaccine is proven safe and effective and is available, will you get vaccinated?” Participants who responded with “definitely not” or “probably not” were categorized as having vaccine non-acceptance, while those who responded with “probably” or “definitely” were categorized as willing to accept the COVID-19 vaccination.

### 3.3. The Uptake Rate of the COVID-19 Vaccines among HCWs

Table 2 presents the characteristics and COVID-19 vaccine uptake rates among HCWs represented in studies contained in this review.

Fifty-two studies reported on COVID-19 vaccination acceptability, intention, and hesitancy. In this review, most of these studies reported HCWs’ hesitation to accept the COVID-19 vaccines on the African continent. A qualitative study conducted by Ashipala and colleagues [63] did not provide information on nurses’ uptake of COVID-19 vaccines.

Twenty-seven studies reported on the intention to accept the COVID-19 vaccine. Intention to accept the vaccine varied dramatically from 21% to 90.1%. Notably, Fares and colleagues [47] found that Egypt (21%) had the lowest intention rate, while Adeniyi and colleagues [52] reported that South Africa (90.1%) had the highest intention rate. Based on the included studies in this review, the intention rate to uptake the COVID-19 vaccine among HCWs was below average [23,25,46,47,54,59,76,77,80,83,88]. Conversely, fourteen studies reported an above-average intention rate [48,49,51,52,55,60,69,71,79,82,88,91,92]. The overall average intention rate for HCWs to uptake the COVID-19 vaccines across all included studies was approximately 52%, indicating a suboptimal level of uptake among this population.

Medical students expressed a lack of willingness to accept the COVID-19 vaccine, with an acceptance rate ranging from 34.7% to 45.4%. A study conducted by Saied and colleagues [83] in Egypt found that only 34.7% of medical students were willing to accept the vaccine, which was disappointing. Most (45.7%) medical students hesitated to accept the vaccine. In addition, 71% intended to take the vaccine but would postpone doing so to wait and observe its effects on those who received it before making a decision themselves.

Twenty-nine studies examined HCWs’ hesitancy towards receiving the COVID-19 vaccine. The degree of hesitancy varied across these studies, ranging from 13.3% to 79%. Fares and colleagues [47] reported the highest VH rate (79%) in Egypt.

**Figure 2:**
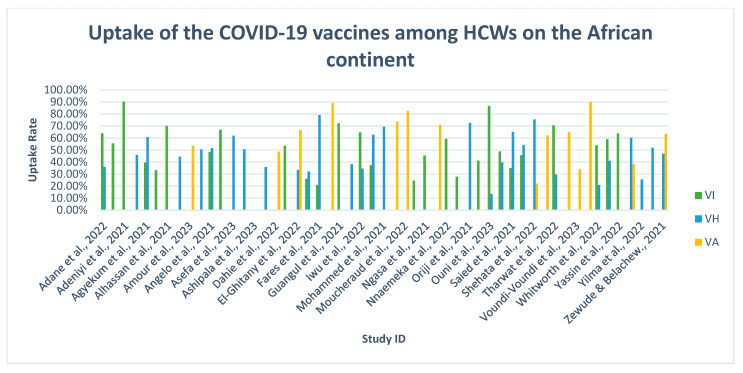
An illustration of COVID-19 vaccine uptake rates among the included studies in Africa [23,47,50,51,52,55,57,59,61,63,65,67,69,71,73,75,77,79,81,82,84,86,88,89,91,92,95].

Subsequent studies reported HCWs’ acceptance towards the COVID-19 vaccines [43,57,65,68,74,75,78,86,87,89,90,93,95]. Among these ten studies, over half of the participants were vaccinated with at least one dose (see Figure 2). A study by Watermeyer and colleagues [90] reported the highest vaccination rate (90%) in South Africa. Additionally, a study conducted in Ethiopia by Zewude and Belachew [95] further depicted the intention to accept the second dose. Approximately 28.3% of HCWs were VH to accept the second dose.

### 3.4. Socio-Demographic Determinants Associated with VA or VH

Table 3 reports various socio-demographic (individual level) factors influencing vaccine uptake. These factors varied across HCWs on the African continent. Twelve socio-demographic factors were associated with vaccine uptake in this review. Seven socio-demographic factors were prominent in influencing vaccine uptake. These included gender, age, level of education, marital status, presence of chronic illness, living area, and cadre. These factors were further divided into two categories, which include COVID-19 vaccine uptake associated with hesitancy and associated with acceptance. Factors associated with COVID-19 vaccine uptake included being male, middle-aged (older than 40), being a physician, and having a tertiary-level education. In contrast, factors associated with hesitancy towards the COVID-19 vaccine were females younger than 40 and having a tertiary education. Interestingly, a tertiary-level education was a significant factor associated with VA and VH among HCWs.

The following factors associated with VA were gender [23,46,56,65,67,72,74,76,77,79,80,87], age [43,46,48,54,56,57,65,74,87,94], education level [43,46,50,52,67,75,78], belonging to religion [48,74], marital status [43,72,76,77,78], being a parent [95], absence of pregnancy [43], presence of chronic illness [43,56,59,77], living area [65,67,77,79], cadre [23,43,48,49,51,53,57,59,61,65,73,79,80,87], and income level [43,46].

In contrast, the following factors were associated with VH, gender [50,55,85,86,89,94], age [50,53,58,64,73,86,94], ethnicity [64], education level [50,55,70,85], religion [71], marital status [58], presence of chronic illness [62], cadre [50,58,64,71,84,93], and income level [58].

### 3.5. Barriers and Facilitators Affecting Vaccine Uptake among HCWs

At the intrapersonal level, three themes emerged: vaccine-related factors, COVID-19, and psychosocial factors. Within the theme of COVID-19 vaccines, ten sub-themes were identified, all acting as barriers to vaccine uptake. The most prominent sub-theme was safety concerns, which was reported as the primary barrier [23,25,43,47,50,51,55,56,57,60,61,65,66,67,68,69,70,72,74,75,76,77,78,81,82,83,84,85,86,88,90,91,92,95]. However, only three studies mentioned confidence in the COVID-19 vaccines, facilitating uptake [47,52,88]. Numerous studies [23,47,55,56,61,66,68,69,70,74,75,77,81,82,85,90,91] highlighted the prevalent mistrust in science among HCWs, often rooted in the belief that the COVID-19 vaccine has not undergone sufficient clinical trials. Concerns about the vaccine’s effectiveness were reported in 16 studies [23,25,65,67,69,70,76,77,78,82,84,85,86,88,92,95], with some expressing doubts about its ability to protect against COVID-19, particularly in Africa. In contrast, only one study reported that the vaccine was effective against COVID-19 [74]. Three studies mentioned that HCWs preferred alternative treatments to the COVID-19 vaccine, such as hydroxychloroquine, azithromycin, and ivermectin [61,81,94]. The subsequent studies reported on other COVID-19 vaccine-related barriers, which included poor vaccine knowledge [66], negative perceptions toward the vaccine [43], preference for waiting for another type of vaccine [70], and not considering the vaccine a priority [70]. Vaccine safety, mistrust in science, and efficacy were major concerns among HCWs within this theme. The following study [95] reported barriers to the uptake of the second vaccine dose, such as discomfort during the first dose and the belief that sufficient immunity had already been acquired.

The second theme in this level was COVID-19, with four sub-themes identified. The perception of susceptibility to contracting COVID-19 among HCWs was mentioned as both a barrier and a facilitator for vaccine uptake. HCWs who perceived themselves to be at a higher risk of contracting COVID-19 [25,47,59,63,88,92] were more willing to get vaccinated compared to those who perceived themselves to have a low risk [23,66,67,78,91]. HCWs who believed they needed the vaccine for protection were more likely to get vaccinated than those who relied on their immune system to prevent infection [65,68,76,77,95]. A prior diagnosis of COVID-19 was mentioned as a barrier to vaccine uptake as some HCWs believed that they had gained natural immunity and did not need the vaccine [23,67,91,92]. Side effects of COVID-19, such as loss of smell and taste, were mentioned as facilitators for vaccine uptake [56].

The final sub-theme at this level was psychosocial factors, which are individual factors that affect vaccine uptake. In separate studies, HCWs with pre-existing health conditions were mentioned as barriers and facilitators [56,59]. Female HCWs planning to conceive were less likely to get vaccinated [67,70,91]. Religious beliefs also played a role as a barrier, with Christian HCWs expressing concerns about the vaccine containing the mark of the beast [55,56,61,66,70,81,95]. Other barriers to uptake at this level included prior adverse reactions to vaccines [23,61], fear of needles and injections [70], and opposition to vaccinations in general [91].

At the interpersonal level, a significant factor relating to influences was discovered. HCWs reported that their relationships with colleagues played a role in encouraging vaccine uptake [63]. HCWs mentioned that their colleagues influenced their decision to get vaccinated. The connection between HCWs and their families also emerged as a crucial sub-theme. The desire to protect their loved ones motivated HCWs to receive the COVID-19 vaccine, as mentioned in eight studies [25,60,72,78,84,88,91,92].

Moreover, one study found that HCWs who had experienced the loss of a loved one due to COVID-19 were more likely to get vaccinated [55]. Within this theme, two barriers were identified. In one study, HCWs expressed the need for permission from their families before getting the COVID-19 vaccine [70]. In another study, HCWs reported facing disapproval from their families regarding the COVID-19 vaccine [66]. The last sub-theme explored religious leaders’ influences on HCWs, indicating that discouragement from religious leaders also acted as a barrier [66].

At the institutional level, there are significant challenges in the environmental structures. One identified barrier is the lack of trust in stakeholders, such as government and pharmaceutical companies [25,43,56,57,68,81,90]. Furthermore, a study [66] found that some HCWs would refuse the vaccine because government officials themselves did not accept it. The accessibility of the vaccine was mentioned as a barrier in four studies [63,65,70,75]. In contrast, one study suggested that the easy availability of the COVID-19 vaccine could be a reason for its uptake [63]. The workplace environment of HCWs also influences vaccine uptake. Lack of support from employers was identified as a barrier, leading HCWs to reject the vaccine [66]. Conversely, another study revealed that some HCWs felt compelled to accept the COVID-19 vaccine to continue working, per their company’s policy [91].

At the community level, a prevailing theme was centred around shared norms and myths. Within this overarching theme, three sub-themes were identified. Multiple studies [52,78,91,92] emphasized that HCWs viewed the uptake of the COVID-19 vaccine as a crucial public health responsibility for ending the pandemic. However, specific barriers to vaccine uptake were also identified. Several studies [23,25,57,61,63,67,70,78] observed that limited access to reliable information hindered the willingness of HCWs to receive the vaccine. Social media emerged as a significant influencer, with seven studies [57,60,63,68,70,72,90] reporting that HCWs subscribed to misinformation or conspiracy theories. These theories included beliefs that the vaccine was intentionally designed to cause harm to people in Africa, sterilize the African population, or even cause COVID-19.

At the policy level, an important theme that emerged was the implementation of COVID-19 policies. Within this theme, two specific sub-themes were identified. The first sub-theme focused on strategies to encourage HCWs to get vaccinated. It was supported by three studies, which highlighted that HCWs would be required to receive the vaccine to travel in the future [47,60,63]. Additionally, two studies indicated that HCWs are willing to accept the COVID-19 vaccine because it is free of charge [74,88]. However, it is worth noting that there is also a barrier at this level. This barrier stems from mandatory vaccination policies, which make HCWs feel coerced into accepting the vaccines [82,89]. HCWs believe they lack control over their health-related behaviours and refuse to be controlled by others, resulting in their rejection of the COVID-19 vaccine. Table 4 summarizes the factors influencing vaccine uptake. 

## 4. Discussion

VH and refusal continue to jeopardize COVID-19 vaccination coverage in LMICs [23]. The fight against COVID-19 requires widespread vaccination uptake and acceptance [96]. In this review, 53 articles were selected and analysed, focusing on the intention, socio-demographical determinants, and factors influencing vaccine uptake. In this review, most studies were conducted in Ethiopia and Nigeria. The intention to take the COVID-19 vaccine is a challenge globally. We found that the proportion of HCWs who intend to take the COVID-19 vaccine was unsatisfactory (52%), with the intention rate ranging from 21% to 90.1%. This finding aligns with a global review by Li and colleagues [97] and Ghare and colleagues [98], who found similar acceptance rates among HCWs ranging from 27.7% to 77.3% and 30% to 98.9% (respectively). HCWs in Africa, particularly in countries such as Egypt, Uganda, and the DRC, seem hesitant about the uptake of the COVID-19 vaccination.

The results pertaining to VH in the studies are likely to be influenced to some extent by the timing of various Information, Education, and Communication (IEC) interventions within the different African countries and vaccine availability at the time of the respective studies. It should also be considered that despite the timing of the studies and vaccine availability in the respective African countries, research findings on vaccine side effects are likely to have played and continue to play a role in VH in particular African countries [99]. Furthermore, as outlined earlier, the previous negative experiences of many African countries with vaccines impact views about the desirability and safety of vaccines [100].

A better understanding of the factors influencing the uptake of COVID-19 vaccines is required to improve vaccine acceptance. Accordingly, this review was conducted using the SEM, which identified several factors that influence the uptake of COVID-19 vaccines. These factors were classified into five levels: intrapersonal, interpersonal, organizational, community, and policy. We found that socio-demographic determinants (intrapersonal level factors) were associated with COVID-19 vaccination. Li and colleagues’ [97] systematic review and Ghare and colleagues’ review [98] aligns with the findings of this scoping review. Socio-demographic determinants associated with COVID-19 vaccine uptake included being male, older age, physician, level of education, and presence of chronic illness. Studies have identified gender differences as a significant cause of VH in low-income countries [56,101]. VA was found to be significantly associated with gender, and specifically the male gender. Naidoo and colleagues’ [102] review reported that men were more accepting of the COVID-19 vaccines among the general African population. This finding is highly noteworthy in African society, where men make most family decisions, regardless of profession or social status [56]. In this review, we found that women were more likely than men to reject the COVID-19 vaccine. While Saied and colleagues [84] noticed that HCWs’ age could explain the difference in uptake; older HCWs appear more accepting due to the prevalence of co-morbidities and a high perceived susceptibility to contracting COVID [99].

Using the SEM, we have identified significant barriers within the five levels. Prominent individual-level barriers include vaccine safety and efficacy concerns and HCWs’ mistrust of science. Contrary to common assumptions that HCWs would have a positive attitude toward COVID-19 vaccines because of their expertise, Verger and colleagues [103] and El-Sokkary and colleagues [46] point out that HCWs are not a homogeneous group and that the vast majority are not immunization experts. Various information sources shape the general public’s vaccine knowledge, influencing vaccination attitudes, perceptions, and uptake [104]. Many studies have shown that individuals who lack adequate knowledge about vaccines or vaccine-preventable diseases (VPDs) are more prone to harbour a negative attitude towards vaccination [105,106]. The development of COVID-19 vaccines exposed a lack of knowledge in immunology among HCWs [46]. Two studies [25,81] cited that HCWs preferred using alternative treatments over accepting the COVID-19 vaccine. According to Oriji and colleagues [81], some (17%) respondents have already taken Hydroxychloroquine and Azithromycin as prophylaxis treatment for COVID-19. Allagoa and colleagues [56] and Oriji and colleagues [81] reported that most respondents who received the COVID-19 vaccine preferred a single-dose vaccine. The number of vaccine doses may have a negative impact on vaccination uptake. Religious beliefs were among the factors associated with vaccine refusal. Studies reviewed [55,56,81] discovered that those of Christian faith were more risk-averse regarding the uptake of the COVID-19 vaccines. However, fatalistic ideas combined with religious beliefs have been found to facilitate questioning about the efficacy of COVID-19 vaccines and that religious fatalism negatively impacts the acceptance of the SARS-CoV-2 vaccine [107].

Misinformation, primarily spread through social media, has fostered distrust in government officials, regulatory agencies, and pharmaceutical companies [102]. The media, particularly social media, has been a significant source of speculation and misinformation about the pandemic and COVID-19 vaccines [108]. According to some HCWs, the media has exaggerated the severity of the side effects of the vaccines [108]. HCWs are a trustworthy source of health information. Their acceptance or rejection of COVID-19 vaccines may impact the broader population’s acceptance and uptake of COVID-19 vaccines [23]. The low intention rate is due to the rapid development of COVID-19 vaccines, concerns about the vaccines’ safety and effectiveness, and cultural and social norms.

On a positive note, our review also identified facilitators at each level. At the intrapersonal level, HCWs’ high perceived susceptibility to COVID-19 and the desire to protect themselves were prominent factors. The African concept of ubuntu, which emphasizes interconnectedness and collective responsibility, influenced COVID-19 vaccine uptake at the interpersonal and community levels. HCWs were eager to receive the vaccine to protect their loved ones and saw it as a public responsibility to end the pandemic.

Governments, public health agencies, and private healthcare systems should collaborate in making educational resources available to inform HCWs about the vaccine’s safety, importance, and the negative consequences of refusing or delaying vaccination [69]. Most studies emphasized how crucial it is for stakeholders to inform and increase HCW awareness of COVID-19 vaccines. It is now up to various stakeholders and policymakers to take effective action to spread as much knowledge as possible among HCWs to increase vaccine acceptance and, thereby, address the pandemic’s detrimental effects on healthcare systems and socio-economic conditions. When tailored education campaigns are targeted to specific attitudes, beliefs, and experiences, they are beneficial [100]. The findings from this review will assist in the roll-out of other vaccination programmes.

### Strengths and Limitations

The majority of articles reviewed adopted a quantitative approach. The present review investigates factors influencing HCWs’ intention and uptake of COVID-19 vaccines. Limitations are inherent in a scoping review approach. Some limitations should be considered in this review. This review did not undertake a quality or risk assessment bias of the included studies. Only studies published in English were considered. There is a bias in the body of literature towards VH. Due to the heterogeneity in the definition and assessment of VH in different studies, not all studies reported VH rates among HCWs. In some studies, the measurement scales used to assess the intention to uptake and VH rates for COVID-19 vaccines were either dichotomous or Likert. The varied sample size would be attributed to selection bias in studies focusing on HCWs. Social desirability on self-reported VH among the HCWs can also not be ruled out. At the time of data collection, some studies did not receive the COVID-19 vaccine. Therefore, intentions and VH may have influenced participants’ responses. The trends in acceptance might have changed after the vaccination programmes were implemented.

## 5. Conclusions

Preventive measures are essential to the global effort to mitigate the pandemic’s consequences. As a result, enormous resources have been dedicated to developing effective and safe COVID-19 vaccines. Using the SEM, this review explored various factors affecting the uptake, allowing for a more comprehensive understanding of vaccine uptake and the development of effective interventions. VI and VH rates vary greatly across countries or regions within the same country. Furthermore, the VI and VH rate is influenced by various factors. Most studies reviewed found significant barriers that affected vaccine uptake on the African continent among HCWs, resulting in a subpar intention to use COVID-19 vaccines. The low level of trust in COVID-19 vaccines and the concerns about the long-term efficacy of the vaccines, as well as the possible long-term side effects associated with the vaccine uptake, play a role in decision-making regarding vaccination. HCWs are influential in informing the general public about vaccines. Therefore, it is crucial to prioritize engagement with key stakeholders to address HCWs’ negative perceptions about vaccines and where they exist in efforts to increase vaccine uptake.

To improve vaccine uptake using the SEM, interventions should target multiple levels simultaneously. At an individual level, understand their concerns and reasons for hesitancy. Provide accurate information to address myths and misconceptions by implementing strategies addressing knowledge gaps and building trust among HCWs. At an organizational level, healthcare facilities should prioritize vaccination by educating staff, offering paid time off for vaccination and side effects, improving access by getting vaccinated as quickly and conveniently as possible, and incentivizing vaccination. They set the culture—if the leadership gets vaccinated, others will follow and leverage social networks and community influencers can have a synergistic effect on increasing vaccine acceptance and uptake. By considering the various levels of influence, the SEM provides a comprehensive framework for understanding and addressing VH and holistically promoting vaccine uptake.

## Figures and Tables

**Table 1:** Illustrates the number of countries reviewed.

Country of Focus	Number of Studies
Ethiopia	12
Nigeria	9
South Africa (SA)	4
Ghana	4
Tanzania	1
Namibia	1
Somalia	2
Egypt	7
Uganda	2
Malawi	1
Zambia	1
Cameroon	2
The Democratic Republic of Congo (DRC)	1
Guinea	1
Sudan	1
Sierra Leone	1
Tunisia	1
Multiple African countries	2

**Table 2:** COVID-19 vaccine uptake rates by author and country.

Author(s) & Publication Year	Country	Vaccine Intention (VI)	Vaccine Hesitant (VH)	Vaccine Acceptance (VA)
**Adane et al., 2022** **[51]**	Ethiopia	64.0%	36.0%	
**Adejumo et al., 2021** **[49]**	Nigeria	55.5%		
**Adeniyi et al., 2021** **[52]**	South Africa	90.1%		
**Aemro et al., 2021** **[53]**	Ethiopia		45.9%	
**Agyekum et al., 2021** **[23]**	Ghana	39.6%	60.7%	
**Ahmed et al., 2021** **[54]**	Ethiopia	33.2%		
**Alhassan et al., 2021** **[55]**	Ghana	70.0%		
**Allagoa et al., 2021** **[56]**	Nigeria		44.5%	
**Amour et al., 2023** **[57]**	Tanzania			53.4%
**Amuzie et al., 2021** **[58]**	Nigeria		50.5%	
**Angelo et al., 2021** **[59]**	Ethiopia	48.4%	51.6%	
**Annan et al., 2021** **[60]**	Ghana	66.9%		
**Asefa et al., 2023** **[61]**	Ethiopia		61.9%	
**Aseneh et al., 2023** **[62]**	Multiple countries Cameroon & Nigeria		50.7%	
**Ashipala et al., 2023** **[63]**	Namibia			
**Berhe et al., 2022** **[64]**	Ethiopia		35.8%	
**Dahie et al., 2022** **[65]**	Somalia			48.7%
**Ekwebene et al., 2021** **[66]**	Nigeria	53.5%		
**El-Ghitany et al., 2022** **[67]**	Egypt		33.5%	66.5%
**El-Sokkary et al., 2021** **[46]**	Egypt	26%	32.1%	
**Fares et al., 2021** **[47]**	Egypt	21%	79%	
**George et al., 2023** **[68]**	South Africa			89%
**Guangul et al., 2021** **[69]**	Ethiopia	72.2%		
**Ibrahim et al., 2023** **[70]**	Somalia		38.2%	
**Iwu et al., 2022** **[71]**	Nigeria	64.6%	34.5%	
**Kanyike et al., 2021** **[72]**	Uganda	37.3%	62.7%	
**Mohammed et al., 2021** **[73]**	Ethiopia		60.3%	
**Mohammed et al., 2023** **[74]**	Ghana			73.6%
**Moucheraud et al., 2022** **[75]**	Malawi			82.5%
**Mudenda et al., 2022** **[76]**	Zambia	24.5%		
**Ngasa et al., 2021** **[77]**	Cameroon	45.4%		
**Niguse et al., 2023** **[78]**	Ethiopia			71%
**Nnaemeka et al., 2022** **[79]**	Nigeria	59.3%		
**Nzaji et al., 2020** **[80]**	The Democratic Republic of Congo	27.7%		
**Oriji et al., 2021** **[81]**	Nigeria		72.5%	
**Orok et al., 2022** **[25]**	Nigeria	41.2%		
**Ouni et al., 2023** **[82]**	Uganda	86.7%	13.3%	
**Robinson et al., 2021** **[83]**	Nigeria	48.8%	39.7%	
**Saied et al., 2021** **[84]**	Egypt	34.9%	65.1%	
**Sharaf et al., 2022** **[85]**	Egypt	45.6%	54.3%	
**Shehata et al., 2022** **[86]**	Egypt		75.5%	22%
**Terefa et al., 2021** **[87]**	Ethiopia			62.1%
**Tharwat et al., 2022** **[88]**	Egypt	70.5%	29.5%	
**Toure et al., 2022** **[43]**	Guinea			65%
**Voundi-Voundi et al., 2023** **[89]**	Cameroon			34%
**Watermeyer et al., 2022** **[90]**	South Africa			90%
**Whitworth et al., 2022** **[91]**	Multiple countries Sierra Leone DRC Uganda	53.9%	21%	
**Wiysonge et al., 2022** **[48]**	South Africa	59%	41%	
**Yassin et al., 2022** **[92]**	Sudan	63.8%		
**Yendewa et al., 2022** **[93]**	Sierra Leone		60.1%	38.3%
**Yilma et al., 2022** **[50]**	Ethiopia		25.5%	
**Zammit et al., 2022** **[94]**	Tunisia		51.9%	
**Zewude & Belachew, 2021** **[95]**	Ethiopia		46.9%	63.4%

**Table 3:** Socio-demographic determinants associated with vaccine uptake.

Factors	Associated with Hesitancy	Associated with Acceptance
**Gender**	Being female [50,55,85,86,89,94]	Being female [47] Being male [23,46,56,65,67,72,74,76,77,79,80,87]
**Age**	Younger [50] <30 years [53,58] <35 years [64] <40 years [73,86,94]	Age [54] >30 years [57] >40 years [65,74,87,94] Older [43,46,48,56]
**Ethnicity**	Amhara [64]	
**Education level**	Tertiary level [50,55,70,86]	Secondary level [43,67] Tertiary level [46,50,52,65,78]
**Religion**	Christian—Pentecostal denomination [71]	Not specified [48] Christian [74]
**Marital status**	Single [58]	Single [72,76] Married [43,77,78]
**Family status**		Being a parent [95]
**Pregnancy status**		Not being pregnant [43]
**Medical condition**	Presence of chronic illness [62]	Presence of chronic illness [43,56,59,77]
**Residential settings**		Not specified [65,79] Rural [67] Urban [77]
**Cadre**	Nurses & midwives [50,58] Physicians [58,84] Medical laboratory technicians [50,64,71] Environmental health specialist [64] Medical students [93]	Not specified [53,73] Nurses & midwives [43,51,65] Physicians [23,48,57,59,61,65,79,80] Clinical health workers [50] Public health specialist [65] Academic staff working in hospitals [87]
**Income level**	Average [58]	Not specified [43,46]

**Table 4:** Factors influencing vaccine uptake.

Table	Factors	Barriers	Facilitators
**Intrapersonal Level**			
**Vaccine related factors**	**Vaccine safety**	Safety concerns [23,25,33,48,50,51,55,56,57,60,61,65,66,67,68,69,70,72,74,75,76,77,78,81,82,83,84,85,86,88,91,92,95]	Confident in the COVID-19 vaccines [47,52,88]
	**Vaccine efficacy**	Concerns about the effectiveness of the vaccine [23,25,65,67,69,70,76,77,78,82,84,85,86,88,92,95]	Belief that the vaccine is effective in protecting against COVID-19 [74]
	**Vaccine knowledge**	Having poor knowledge [66]	
	**Vaccine perception**	Having a negative perception [43]	
	**Vaccine preference**	Prefer to wait for another type of COVID-19 vaccine [70]	
	**Vaccine necessity**	Not a priority [70]	
	**Vaccine experiences**	Experiences of discomfort while receiving the first dose [95]	
	**Vaccine immunity against COVID-19**	Sufficient immunity with the first dose [95]	
	**Vaccine vs. alternative treatment**	Preferred alternative treatment to the COVID-19 vaccine [61,81,95]	
	**Vaccine development**	Mistrust in science [23,47,55,56,61,66,68,69,70,74,75,77,81,82,85,90,91]	
**COVID-19**	**Diagnosis of COVID**	Prior diagnosis [23,67,91,92]	
	**Susceptibility of contracting COVID**	Low perceived susceptibility [23,66,67,78,91]	High perceived susceptibility [25,47,59,63,87,92]
	**Side-effects of COVID**		Previous history of loss of smell & taste [56]
	**Protection against COVID**	Belief in one’s immune system [65,68,76,77,95]	Requires the vaccine to protect oneself [60,72,74,78,84,88,91]
**Psychosocial factors**	**Chronic illness**	Presence of chronic illness [56]	Presence of chronic illness [59]
	**Family planning**	Planning pregnancy [67,70,91]	
	**Religion**	Religious beliefs [55,56,61,66,70,81,95]	
	**Experiences with vaccines**	Prior adverse reactions to vaccines [2,61]	
		Fear of needles & injections [70]	
		Against vaccinations in general [91]	
**Interpersonal Level**			
**Influences**	**Relationship with colleagues**		Being influenced by colleagues [63]
	**Relationship with family**	Requires permission from their family before taking the COVID-19 vaccine [70]	
		Disapproval from family [66]	
			Desire to protect loved ones [25,60,72,78,84,88,91,92]
			Loss of someone to COVID-19 [55]
	**Relationship with religious leaders**	Discouragement from Religious leaders [66]	
**Organizational Level**			
**Institutional structures**	**Government & stakeholders**	Lack of trust [25,43,56,57,68,81,90]	
		Government officials not accepting vaccine uptake [66]	
	**Vaccine accessibility**	COVID-19 vaccine inaccessible [63,65,70,75]	COVID-19 vaccine accessible [63]
**Workplace environment**	**Company policy**		To keep working [91]
	**Leadership & support**	Lack of support by employer [66]	
**Community Level**			
**Shared norms & myths**	**Public health responsibility**		To end the pandemic [52,78,91,92]
	**Access to information**	Lack of information [23,25,57,61,63,67,70,78]	
	**Social media**	Subscribing to misinformation or conspiracies [57,60,63,68,70,72,90]	
**Policy Level**			
**Vaccination policies**	**Travel requirements**		Requires the vaccine for future travel [47,60,63]
	**Vaccination cost**		Vaccines are provided free of charge [74,88]
	**Mandatory policies**	Feeling coerced into accepting vaccines [82,89]	

## Data Availability

Not applicable.

## Supplementary Materials

The following supporting information can be downloaded at: https://www.mdpi.com/article/10.3390/vaccines11091491/s1, Supplementary Materials S1: PRISMA-ScR-Fillable-Checklist—HCWs.

## Appendix A. Search Strategy

**Table A1:** WorldCat Discovery search strategy.

Search Terms	Filters	Results
kw: COVID-19 vaccine AND	Format: Article	16
kw: Vaccine Hesitancy AND		
kw: Vaccine acceptance AND	Language: English	
kw: Africa AND		
kw: Healthcare workers	Publication Year: 2020–2023	

**Table A2:** PubMed search strategy.

Search Number	Query	Filters	Search Details	Results	Time
**10**	((((((COVID-19 vaccines[MeSH Terms]) AND (COVID-19)) AND (vaccines)) OR (covid vaccines)) OR (intention)) OR (vaccine hesitancy)) AND (vaccine acceptance) AND (healthcare workers) AND (Africa)	Full text, Humans, English, from 2020–2023	(((“covid 19 vaccines” [MeSH Terms] AND (“covid 19” [All Fields] OR “covid 19” [MeSH Terms] OR “covid 19 vaccines” [All Fields] OR “covid 19 vaccines” [MeSH Terms] OR “covid 19 serotherapy” [All Fields] OR “covid 19 nucleic acid testing” [All Fields] OR “covid 19 nucleic acid testing” [MeSH Terms] OR “covid 19 serological testing” [All Fields] OR “covid 19 serological testing” [MeSH Terms] OR “covid 19 testing” [All Fields] OR “covid 19 testing” [MeSH Terms] OR “sars cov 2” [All Fields] OR “sars cov 2” [MeSH Terms] OR “severe acute respiratory syndrome coronavirus 2” [All Fields] OR “ncov” [All Fields] OR “2019 ncov” [All Fields] OR ((“coronavirus” [MeSH Terms] OR “coronavirus” [All Fields] OR “cov” [All Fields]) AND 2019/11/01:3000/12/31[Date—Publication])) AND (“vaccin” [Supplementary Concept] OR “vaccin” [All Fields] OR “vaccination” [MeSH Terms] OR “vaccination” [All Fields] OR “vaccinable” [All Fields] OR “vaccinal” [All Fields] OR “vaccinate” [All Fields] OR “vaccinated” [All Fields] OR “vaccinates” [All Fields] OR “vaccinating” [All Fields] OR “vaccinations” [All Fields] OR “vaccinations” [All Fields] OR “vaccinator” [All Fields] OR “vaccinators” [All Fields] OR “vaccines” [All Fields] OR “vaccined” [All Fields] OR “vaccines” [MeSH Terms] OR “vaccines” [All Fields] OR “vaccine” [All Fields] OR “vaccins” [All Fields])) OR ((“sars cov 2” [MeSH Terms] OR “sars cov 2” [All Fields] OR “covid” [All Fields] OR “covid 19” [MeSH Terms] OR “covid 19” [All Fields]) AND (“vaccin” [Supplementary Concept] OR “vaccin” [All Fields] OR “vaccination” [MeSH Terms] OR “vaccination” [All Fields] OR “vaccinable” [All Fields] OR “vaccinal” [All Fields] OR “vaccinate” [All Fields] OR “vaccinated” [All Fields] OR “vaccinates” [All Fields] OR “vaccinating” [All Fields] OR “vaccinations” [All Fields] OR “vaccinations” [All Fields] OR “vaccinator” [All Fields] OR “vaccinators” [All Fields] OR “vaccines” [All Fields] OR “vaccined” [All Fields] OR “vaccines” [MeSH Terms] OR “vaccines” [All Fields] OR “vaccine” [All Fields] OR “vaccins” [All Fields])) OR (“intention” [MeSH Terms] OR “intention” [All Fields] OR “intent” [All Fields] OR “intentions” [All Fields] OR “intentional” [All Fields] OR “intentioned” [All Fields] OR “intents” [All Fields]) OR (“vaccination hesitancy” [MeSH Terms] OR (“vaccination” [All Fields] AND “hesitancy” [All Fields]) OR “vaccination hesitancy” [All Fields] OR (“vaccine” [All Fields] AND “hesitancy” [All Fields]) OR “vaccine hesitancy” [All Fields])) AND ((“vaccin” [Supplementary Concept] OR “vaccin” [All Fields] OR “vaccination” [MeSH Terms] OR “vaccination” [All Fields] OR “vaccinable” [All Fields] OR “vaccinal” [All Fields] OR “vaccinate” [All Fields] OR “vaccinated” [All Fields] OR “vaccinates” [All Fields] OR “vaccinating” [All Fields] OR “vaccinations” [All Fields] OR “vaccinations” [All Fields] OR “vaccinator” [All Fields] OR “vaccinators” [All Fields] OR “vaccines” [All Fields] OR “vaccined” [All Fields] OR “vaccines” [MeSH Terms] OR “vaccines” [All Fields] OR “vaccine” [All Fields] OR “vaccins” [All Fields]) AND (“accept” [All Fields] OR “acceptabilities” [All Fields] OR “acceptability” [All Fields] OR “acceptable” [All Fields] OR “acceptably” [All Fields] OR “acceptance” [All Fields] OR “acceptances” [All Fields] OR “acceptation” [All Fields] OR “accepted” [All Fields] OR “accepter” [All Fields] OR “accepters” [All Fields] OR “accepting” [All Fields] OR “accepts” [All Fields])) AND (“health personnel” [MeSH Terms] OR (“health” [All Fields] AND “personnel” [All Fields]) OR “health personnel” [All Fields] OR (“healthcare” [All Fields] AND “workers” [All Fields]) OR “healthcare workers” [All Fields]) AND (“africa” [MeSH Terms] OR “africa” [All Fields] OR “africa s” [All Fields] OR “africas” [All Fields])) AND ((fft[Filter]) AND (humans[Filter]) AND (english[Filter]) AND (2020:2023[pdat]))	41	9:28:21

**Table A3:** ProQuest search strategy.

Set No.	Searched for	Databases	Results
S9	((factors associated with covid- 19 vaccine hesitancy among HCWs in Africa) AND (location.exact(“Africa” OR “South Africa” OR “Nigeria” OR “Ethiopia” OR “Egypt” OR “Ghana” OR “Uganda” OR “Central Africa” OR “North Africa” OR “Sierra Leone” OR “West Africa” OR “Zambia” OR “Zimbabwe” OR “Burkina Faso” OR “Cape Town South Africa” OR “Congo-Democratic Republic of Congo” OR “East Africa” OR “Eastern Cape South Africa” OR “Kano Nigeria” OR “Kenya” OR “Malawi” OR “Mozambique”) AND at.exact(“Article”) AND la.exact(“ENG”) AND PEER(yes))) AND ((factors associated with covid-19 vaccine uptake among HCWs in Africa) AND (location.exact(“Africa” OR “South Africa” OR “Nigeria” OR “Ethiopia” OR “Egypt” OR “Ghana” OR “Uganda” OR “Central Africa” OR “North Africa” OR “Sierra Leone” OR “West Africa” OR “Zambia” OR “Zimbabwe” OR “Burkina Faso” OR “Cape Town South Africa” OR “Congo-Democratic Republic of Congo” OR “East Africa” OR “Eastern Cape South Africa” OR “Kano Nigeria” OR “Kenya” OR “Malawi” OR “Mozambique”) AND at.exact(“Article”) AND la.exact(“ENG”) AND PEER(yes)))	Coronavirus Research Database, Ebook Central, Health Research Premium Collection, Publicly Available Content Database These databases are searched for part of your query.	48

## Appendix B

**Table A4:** Included Study Characteristics.

Author(s) & Publication Year	Country & Data Collection Period	Methodology
Adane et al., 2022 [51]	Ethiopia May 2021	**Study design:** A quantitative cross-sectional study **Population target:** Physicians Medical Laboratory Technicians Nurses & Midwives Pharmacists Radiologists Anaesthesiologists Public Health Specialist Non-medical Auxiliary Staff **Sample size**: 404 **Measurement scale:** Likert scale
Adejumo et al., 2021 [49]	Nigeria October 2020	**Study design:** A quantitative cross-sectional study **Population target:** Physicians Nurses Medical Laboratory Technicians Pharmacists Physiotherapists Other **Sample size:** 1470 **Measurement scale:** Dichotomous scale
Adeniyi et al., 2021 [52]	South Africa November to December 2020	**Study design:** A quantitative cross-sectional study **Population target:** Physicians Pharmacists Nurses Allied Health Professionals Support Staff **Sample size:** 1380 **Measurement scale:** Dichotomous scale
Aemro et al., 2021 [53]	Ethiopia May to June 2021	**Study design:** A quantitative cross-sectional study **Population target:** Physicians Pharmacists Nurses Allied Health Professionals Support Staff **Sample size:** 418 **Measurement scale:** Dichotomous scale
Agyekum et al., 2021 [23]	Ghana January to February 2021	**Study design:** A quantitative cross-sectional study **Population target:** Nurses & Midwives Allied Health Professionals Physicians **Sample size:** 234 **Measurement scale:** Dichotomous scale
Ahmed et al., 2021 [54]	Ethiopia January to March 2021	**Study design:** A quantitative cross-sectional study **Population target:** Nurses & Midwives Psychiatrists Optometrists Physicians Health Officers Anaesthetics Medical Laboratory Technicians Radiologists Physiotherapists Pharmacists Other **Sample size:** 409 **Measurement scale:** Dichotomous scale
Alhassan et al., 2021 [55]	Ghana September to October 2020	**Study design:** A quantitative cross-sectional study **Population target:** Pharmacists Other **Sample size:** 1605 **Measurement scale:** Dichotomous scale
Allagoa et al., 2021 [56]	Nigeria April 2021	**Study design:** A quantitative cross-sectional study **Population target:** Physicians **Sample size:** 182 **Measurement scale:** Dichotomous scale
Amour et al., 2023 [57]	Tanzania October to November 2021	**Study design:** A mixed-method study **Population target:** Physicians Nurses & Midwives Pharmacists Medical Laboratory Technicians Administrative Staff Other **Sample size:** 1368
Amuzie et al., 2021 [58]	Nigeria March 2021	**Study design:** A quantitative cross-sectional study **Population target:** Physicians Nurses Pharmacists Medical Laboratory Technicians Administrative Staff Allied Health Professionals **Sample size:** 422 **Measurement scale:** Dichotomous scale
Angelo et al., 2021 [59]	Ethiopia March 2021	**Study design:** A quantitative cross-sectional study **Population target:** Physicians Nurses & Midwives Medical Laboratory Technicians Pharmacist **Sample size:** 405 **Measurement scale:** **Dichotomous scale**
Annan et al., 2021 [60]	Ghana	**Study design:** A quantitative cross-sectional study **Population target:** Junior Physicians **Sample size:** 305 **Measurement scale:** Dichotomous scale
Asefa et al., 2023 [61]	Ethiopia July to August 2021	**Study design:** A quantitative cross-sectional study **Population target:** Nurses & Midwives Physicians Medical Laboratory Technicians Pharmacists **Sample size:** 421 **Measurement scale:** Dichotomous scale
Aseneh et al., 2023 [62]	Multiple countries Cameroon & Nigeria May to June 2021	**Study design:** A quantitative cross-sectional study **Population target:** Physicians Nurses & Midwives Administrative Staff Paramedics Pharmacists CHWs Dentists Medical Laboratory Technicians Nurse Assistants Public Health Specialist Physiotherapists Radiologists Other **Sample size:** 598 **Measurement scale:** Dichotomous scale
Ashipala et al., 2023 [63]	Namibia September to October 2021	**Study design:** A qualitative study **Population target:** Nurses **Sample size:** 15
Berhe et al., 2022 [64]	Ethiopia July 2022	**Study design:** A quantitative cross-sectional study **Population target:** Nurses & Midwives Physicians Medical Laboratory Technicians Pharmacist Psychiatrist Environmental Health Specialist Public Health Specialist Others **Sample size:** 403 **Measurement scale:** Dichotomous scale
Dahie et al., 2022 [65]	Somalia December 2021 to February 2022	**Study design:** A quantitative cross-sectional study **Population target:** Nurses & Midwives Physicians Medical Laboratory Technicians Public Health Specialist Dentist Pharmacist CHWs Nutritionists Other **Sample size:** 1281 **Measurement scale:** Dichotomous scale
Ekwebene et al., 2021 [66]	Nigeria	**Study design:** A quantitative cross-sectional study **Population target** Physicians Nurses Public Health Specialist Radiologist Dentists Optometrist Medical Laboratory Technicians Pharmacists Physiotherapist Cleaners **Sample size:** 445 **Measurement scale:** Dichotomous scale
El-Ghitany et al., 2022 [67]	Egypt January to June 2021	**Study design:** A quantitative cross-sectional study **Population target:** Physicians Nurses Pharmacist Other **Sample size**: 2919 **Measurement scale:** Dichotomous scale
El-Sokkary et al., 2021 [46]	Egypt January 2021	**Study design:** A quantitative cross-sectional study **Population target:** Physicians Dentists Pharmacists Others **Sample size:** 308 **Measurement scale:** Likert scale
Fares et al., 2021 [47]	Egypt December 2020 to January 2021	**Study design:** A quantitative cross-sectional study **Population target:** Physicians Nurses Pharmacists Dentists Physiotherapists **Sample size:** 385 **Measurement scale:** Likert scale
George et al., 2023 [68]	South Africa August to October 2022	**Study design:** A mixed-method study **Population target:** Nurses Physicians Allied Health Professionals Dentists/Dental Hygienists Paramedics Pharmacists **Sample size:** 7763 **Measurement scale:** Dichotomous scale
Guangul et al., 2021 [69]	Ethiopia	**Study design:** A quantitative cross-sectional study **Population target:** Health Officer/Clinical officer Medical Laboratory Technicians Nurses Pharmacists Physicians Other **Sample size:** 668 **Measurement scale:** Dichotomous scale
Ibrahim et al., 2023 [70]	Somalia February to March 2022	**Study design:** A quantitative cross-sectional study **Population target:** Nurses & Midwives Physicians Radiologists Medical Laboratory Technicians **Sample size:** 1476 **Measurement scale:** Dichotomous scale
Iwu et al., 2022 [71]	Nigeria September to October 2021	**Study design:** A quantitative cross-sectional study **Population target:** Nurses & Midwives Physicians Medical Laboratory Technicians Pharmacists **Sample size:** 347 **Measurement scale:** Dichotomous scale
Kanyike et al., 2021 [72]	Uganda March 2021	**Study design:** A quantitative cross-sectional study **Population target:** Medical students **Sample size:** 600 **Measurement scale:** Dichotomous scale
Mohammed et al., 2021 [73]	Ethiopia March to July 2021	**Study design:** A quantitative cross-sectional study **Population target:** Nurses & Midwives Physicians Medical Laboratory Technicians Anaesthetic Technicians Pharmacists Radiologists **Sample size:** 614 **Measurement scale:** Dichotomous scale
Mohammed et al., 2023 [74]	Ghana	**Study design:** A quantitative cross-sectional study **Population target:** Physicians Allied Health Professionals Auxiliary Employees **Sample size:** 424 **Measurement scale:** Dichotomous scale
Moucheraud et al., 2022 [75]	Malawi March to May 2021	**Study design:** A quantitative cross-sectional study **Population target:** Physicians Medical Assistants Nurses HIV Diagnostic Assistants Health Surveillance Assistants Patient Supporter Data Clerks **Sample size:** 400 **Measurement scale:** Dichotomous scale
Mudenda et al., 2022 [76]	Zambia February to April 2021	**Study design:** A quantitative cross-sectional study **Population target:** Pharmacy students **Sample size:** 326 **Measurement scale:** Dichotomous scale
Ngasa et al., 2021 [77]	Cameroon April to June 2021	**Study design:** A quantitative cross-sectional study **Population target:** Physicians Medical Students Nurses Medical Laboratory Technicians Public Health Specialist Pharmacists **Sample size:** 371 **Measurement scale:** Dichotomous scale
Niguse et al., 2023 [78]	Ethiopia October to November 2021	**Study design:** A quantitative cross-sectional study **Population target:** Nurses & Midwives Physicians Radiologists Public Health Specialist Pharmacists **Sample size:** 390 **Measurement scale:** Dichotomous scale
Nnaemeka et al., 2022 [79]	Nigeria September 2021 & March 2022	**Study design:** A quantitative cross-sectional study **Population target:** Nurses & Midwives Physicians Pharmacists Medical Laboratory Technicians Radiologists Administrative Staff Physiotherapists **Sample size:** 1268 **Measurement scale:** Dichotomous scale
Nzaji et al., 2020 [80]	The Democratic Republic of Congo March to April 2020	**Study design:** A quantitative cross-sectional study **Population target:** Physicians Nurses Other **Sample size:** 613 **Measurement scale:** Dichotomous scale
Oriji et al., 2021 [81]	Nigeria April 2021	**Study design:** A quantitative cross-sectional study **Population target:** Nurses Pharmacists Medical Laboratory Technicians Non-clinical officers **Sample size:** 182 **Measurement scale:** Likert scale
Orok et al., 2022 [25]	Nigeria May to June 2021	**Study design:** A quantitative cross-sectional study **Population target:** Medical students **Sample size:** 233 **Measurement scale:** Likert scale
Ouni et al., 2023 [82]	Uganda	**Study design:** A mixed-method study **Population target:** Nurses & Midwives Physicians Environmental Health Specialist Medical Laboratory Technicians **Sample size:** 346
Robinson et al., 2021 [83]	Nigeria December 2020 to January 2021	**Study design:** A quantitative cross-sectional study **Population target:** Ancillary Support Staff Dental Technicians Physicians Medical Laboratory Technicians Medical Consultant Nurses & Midwives Optometrists Pharmacist Physiotherapists Primary Healthcare Worker Radiologists **Sample size:** 1094 **Measurement scale:** Likert scale
Saied et al., 2021 [84]	Egypt January 2021	**Study design:** A quantitative cross-sectional study **Population target:** Medical students **Sample size:** 2133 **Measurement scale:** Likert scale
Sharaf et al., 2022 [85]	Egypt August to October 2021	**Study design:** A quantitative cross-sectional study **Population target:** Dental teaching staff **Sample size:** 171 **Measurement scale:** Likert scale
Shehata et al., 2022 [86]	Egypt March to May 2021	**Study design:** A quantitative cross-sectional study **Population target:** Physicians **Sample size:** 1268 **Measurement scale:** Dichotomous scale
Terefa et al., 2021 [87]	Ethiopia June 2021	**Study design:** A quantitative cross-sectional study **Population target:** Nurses & Midwives Physicians Medical Laboratory Technicians Pharmacists Anaesthetists Psychiatrist Dentists Public Health Specialist Other **Sample size:** 522 **Measurement scale:** Dichotomous scale
Tharwat et al., 2022 [88]	Egypt August to September 2021	**Study design:** A quantitative cross-sectional study **Population target:** Nurses & Midwives Physicians Administrative Staff Security Officers Radiologist Medical Laboratory Technicians Pharmacists Dentist **Sample size:** 455 **Measurement scale:** Likert scale
Toure et al., 2022 [43]	Guinea March to August 2021	**Study design:** A mixed-method study **Population target:** General adult population & HCW Nurses & Midwives Medical Laboratory Technicians Physicians **Sample size:** 7210 (HCWs-3547) **Measurement scale:** Dichotomous scale
Voundi-Voundi et al., 2023 [89]	Cameroon January to March 2022	**Study design:** A quantitative cross-sectional study **Population target:** Nurses & Midwives Physicians Administrative Staff **Sample size**: 360
Watermeyer et al., 2022 [90]	South Africa September to November 2021	**Study design:** A qualitative study **Population target:** CHW **Sample size:** 20
Whitworth et al., 2022 [91]	Multiple countries Sierra Leone DRC Uganda April to October 2021	**Study design:** A quantitative cross-sectional study **Population target:** Physicians Nurses & Midwives Clinical Support Staff Medical Laboratory Technicians Pharmacist Non-clinical support staff **Sample size:** 543 **Measurement scale:** Likert scale
Wiysonge et al., 2022 [48]	South Africa March to May 2021	**Study design:** A quantitative cross-sectional study **Population target:** Admin Support Nurses Other HCWs Physicians **Sample size:** 395 **Measurement scale:** Likert scale
Yassin et al., 2022 [92]	Sudan April 2021	**Study design:** A quantitative cross-sectional study **Population target:** Physicians Pharmacist Nurses Medical Laboratory Technicians Administrators Others **Sample size:** 400 **Measurement scale:** Dichotomous scale
Yendewa et al., 2022 [93]	Sierra Leone January to March 2022	**Study design:** A quantitative cross-sectional study **Population target:** Physicians Medical Students Pharmacists Nurses Nursing Students **Sample size**: 592 **Measurement scale:** Likert scale
Yilma et al., 2022 [50]	Ethiopia February to April 2021	**Study design:** A quantitative cross-sectional study **Population target:** Nurses & Midwives Physicians Medical Laboratory Technicians Pharmacists Cleaners Others **Sample size**: 1314 **Measurement scale:** Dichotomous scale
Zammit et al., 2022 [94]	Tunisia January 2021	**Study design:** A quantitative cross-sectional study **Population target:** Physicians Dentists Pharmacists Paramedical professionals **Sample size**: 493 **Measurement scale:** Dichotomous scale
Zewude & Belachew, 2021 [95]	Ethiopia June 2021	**Study design:** A quantitative cross-sectional study **Population target:** Physicians Health officer Administrative Staff Nurse Medical Laboratory Technician Pharmacist Others **Sample size:** 232 **Measurement scale:** Dichotomous scale
